# Cadherin-11 Influences Differentiation in Human Mesenchymal Stem Cells by Regulating the Extracellular Matrix Via the TGFβ1 Pathway

**DOI:** 10.1093/stmcls/sxac026

**Published:** 2022-04-13

**Authors:** Fiona R Passanha, Thomas Geuens, Vanessa L S LaPointe

**Affiliations:** MERLN Institute for Technology-Inspired Regenerative Medicine, Maastricht University, Maastricht, The Netherlands; MERLN Institute for Technology-Inspired Regenerative Medicine, Maastricht University, Maastricht, The Netherlands; MERLN Institute for Technology-Inspired Regenerative Medicine, Maastricht University, Maastricht, The Netherlands

**Keywords:** adipogenic differentiation, fibronectin, SSMAD2/3, type VI collagen

## Abstract

For regenerative medicine, directing stem cell fate is one of the key aims. Human mesenchymal stem cells (hMSCs) are versatile adult stem cells that have been proposed for several clinical applications, making directing their fate of utmost importance. For most clinical applications, their differentiation toward the adipogenic lineage is an undesired outcome. Understanding the mechanisms that regulate hMSC commitment toward the adipogenic lineage might help open up new avenues for fine-tuning implanted hMSCs for regenerative medicine applications. We know that cadherin-11 is required for hMSC commitment to the adipogenic lineage; therefore, we sought to investigate the mechanisms through which cadherin-11 regulates adipogenic differentiation. We observed that hMSCs lacking cadherin-11 had decreased expression of type VI collagen and increased expression of fibronectin. We provide evidence of increased transforming growth factor beta 1 and the subsequent translocation of phosphorylated SMAD2/3 into the nucleus by cells that lack cadherin-11, which could be attributed to the changes in extracellular matrix composition. Taken together, our study implicates cadherin-11 in regulating extracellular matrix production and thereby helping improve cell- and material-based regenerative medicine approaches.

## Graphical Abstract

**Graphical Abstract:**
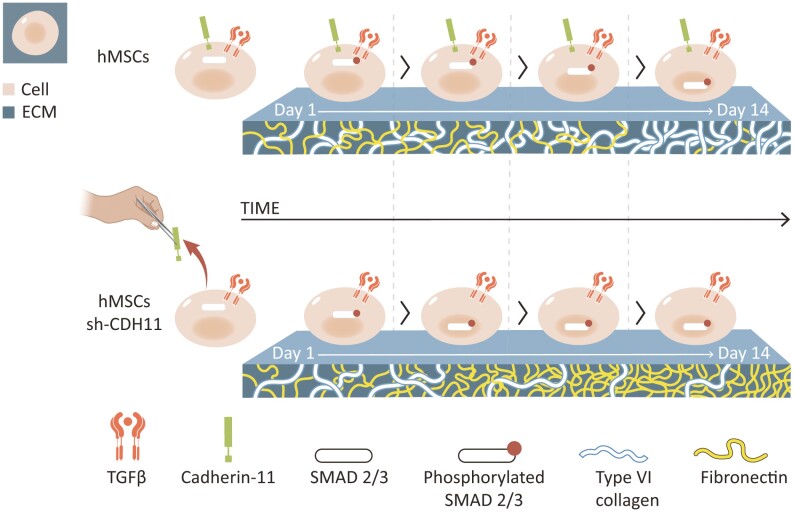

**Significance Statement**

Cadherin-11, a cell adhesion molecule, is important in regulating cellular differentiation. Controlling the fate of human mesenchymal stem cells is of great interest to regenerative medicine. This study aims to understand the mechanisms regulating human mesenchymal stem cell commitment by providing evidence to explain how knocking down cadherin-11 leads to changes in their adipogenic differentiation potential. The importance of the extracellular matrix in influencing cell behavior is unquestionable, and we demonstrate that knocking down cadherin-11 changes the extracellular matrix composition via the transforming growth factor β1 pathway, thereby affecting cell differentiation.

## Introduction

For regenerative medicine, directing stem cell fate is one of the key aims. Studying stem cell communication on a cellular level provides insights into how the human body forms tissues and how it functions, which in turn helps the field build highly developed tissues. Human mesenchymal stem cells (hMSCs) are versatile adult stem cells that have been proposed for several clinical applications, therefore, their preferential fate commitment to various cells of the mesodermal lineage is of utmost interest.^1-3^ However, a full understanding of the underlying mechanisms that govern the fate commitment of hMSCs is necessary to develop better clinical applications of MSCs in regenerative medicine, but remains limited to date.

Cadherin-11 is a cell adhesion molecule expressed by hMSCs, and we recently reported that it is crucial for their commitment toward the adipogenic lineage.^4^ Other studies have also implicated cadherin-11 in hMSC differentiation.^5,6^ HMSCs have shown their potential for the treatment of type 2 diabetes, bone and cartilage disorders, among many others, all of which consider commitment toward the adipogenic lineage an undesired outcome.^7-9^ This is because adipogenic differentiation of hMSCs occurs at the expense of osteogenic and chondrogenic lineage specificity. Adipogenic differentiation is a highly complex process; although the cascade of transcriptional events that leads to adipogenic differentiation is known, the molecular basis of the adipogenic differentiation pathway needs to be better understood in order to find strategies to control it. Understanding the mechanisms that regulate hMSC fate commitment toward the adipogenic lineage might help open up new avenues for fine-tuning implanted hMSCs for regenerative medicine applications.

To this end, we set out to determine the mechanisms through which the knockdown of cadherin-11 disrupts the adipogenic differentiation potential of hMSCs. Cadherin-11 has previously been linked to tissue fibrosis, which is the excessive deposition of extracellular matrix (ECM) components.^10,11^ A recent study also showed that human fibroblasts lacking cadherin-11 had reduced collagen and elastin content.^12^ Then there are numerous studies that indicate that ECM is key to fate commitment.^13,14^ Linking these together, we hypothesized that the cadherin-11 knockdown could alter the ECM in hMSCs by altering the crosstalk between various pathways, thereby disrupting their differentiation toward the adipogenic lineage.

In this study, we not only provide evidence that cadherin-11 knockdown affects collagen expression but also for the first time show that it affects fibronectin expression. We also identified a possible crosstalk with the transforming growth factor beta 1 (TGFβ1) pathway through which cadherin-11 modulates the ECM. By implicating cadherin-11 in the regulation of the ECM, we add to the evidence of the cadherin-integrin crosstalk mechanism.

## Materials and Methods

### Cell Culture

Bone marrow–derived hMSCs (PromoCell) derived from a 65-year-old Caucasian male donor obtained at passage 1 were maintained in growth medium composed of minimum essential medium α (Gibco) supplemented with 10% (v/v) fetal bovine serum (FBS). The medium was changed every second day, and the cells were maintained at 37 °C in 5% CO_2_ in a humidified incubator. Upon reaching 80% confluence, cells were trypsinized in 0.05% trypsin-EDTA and replated for continuous passage. The cells were used at passage 5 for all experiments.

### ShRNA Lentiviral Transduction

The plasmid pLKO.1 containing short hairpin RNA (shRNA) sequences targeting cadherin-11 was obtained from Sigma-Aldrich together with a scrambled negative control. These plasmids were co-transfected with third generation lentiviral packaging and envelope vectors; pMD2.G, pRSV-Rev, and pMDLg/pRRE (Addgene plasmid #12259, #12253, and #12251, respectively, which were gifts from Didier Trono^15^), into HEK-293T cells using PEIpro (VWR) transfection reagent. Lentiviral particles were harvested 48 and 72 h after transfection. The harvested viral supernatant was filtered and stored at −80 °C until use. Five milliliters of viral supernatant were used to transduce hMSCs seeded at 5000 cells/cm^2^ in a T225 flask and incubated for 48 h. After transduction, positive cells were selected with 2 µg/mL puromycin dihydrochloride (Sigma-Aldrich) in growth media for 7 days and were then used for subsequent experiments.

### Induction and Evaluation of Adipogenic Differentiation

HMSCs were seeded at 10 000 cells/cm^2^ and expanded to confluence prior to differentiation. To induce adipogenic differentiation, adipogenic inductive medium composed of Dulbecco’s modified Eagle medium (high glucose, no sodium pyruvate; Gibco) supplemented with 10% FBS, 40 mM indomethacin, 83 mM 3-isobutyl-1-methylxanthine, 10 mg/mL insulin, and 0.1 mM dexamethasone was added to the cells and refreshed every second day. The cultures were maintained for 21 days, after which they were evaluated by Oil Red O staining. Cells were washed twice with phosphate-buffered saline (PBS), fixed in 4% (w/v) paraformaldehyde for 15 minutes at ambient temperature, and washed 3 times with distilled water. The intracellular lipid accumulation was stained with 0.2% (w/v) Oil Red O solution in 60% isopropanol for 15 minutes and images were acquired on a Nikon eclipse TS100 inverted microscope. The lipid droplets were quantified by extracting the Oil Red O from the stained cells. Cells stained with Oil Red O after 21 days in the adipogenic inductive medium were incubated with 4% (v/v) Igepal (Sigma) in isopropanol for 15 minutes by shaking at ambient temperature. Absorbance was measured at 510 nm using a ClarioStar plate reader (BMG LabTech).

### Western Blotting

HMSCs were lysed in radioimmunoprecipitation assay (RIPA) buffer supplemented with 1 tablet per 10 mL RIPA of both protease inhibitor (Sigma-Aldrich) and phosphatase inhibitor (Thermo Fisher Scientific). The lysate was incubated on ice with constant mixing for 30 minutes, followed by sonication using Q700 Sonicator (Qsonica) on ice 3 times for 5 sec with 10% amplitude and 30 sec between each cycle, and finally centrifuged at 16 000 × *g* for 20 minutes at 4 °C. Total protein concentration was measured using the Pierce BCA protein assay kit (Thermo Fisher Scientific). For separation, 20 μg of total protein lysate was supplemented with Laemmli buffer, reduced with 5% (v/v) 2-mercaptoethanol (Sigma-Aldrich) and separated on a 4-15% TGX gel (Bio-Rad) followed by transferring for 90 minutes to a PVDF membrane (Bio-Rad) using the wet transfer method. Membranes were blocked in 5% (w/v) milk in Tris-buffered saline (TBS) with 0.01% (v/v) Tween-20 for 60 minutes before overnight incubation at 4 °C with primary antibodies diluted in blocking buffer. The membranes were washed 3 times and incubated with secondary antibodies in blocking buffer for 2 h at ambient temperature. Primary antibodies were against: type VI collagen (rabbit clone, 1:1000; Genetex, GTX109963), fibronectin (rabbit clone, 1:1000; Novus Biologicals, NBP1-91258), cadherin-11 (rabbit clone, 1:1000; Thermo Fisher Scientific, 71-7600), or GAPDH (mouse clone, 1:1000; Santa Cruz Biotechnology, SC-365062). Secondary antibodies used were: IRDye 680RD goat anti-mouse IgG or IRDye 800CW donkey anti-rabbit IgG (both 1:15,000; LI-COR Biotechnology). The membranes were imaged on an Odyssey infrared imaging system (LI-COR Biotechnology). Band intensities were determined by quantifying the mean pixel gray values using the ImageJ 1.52b software.

### Immunofluorescence

HMSCs were washed twice with PBS and fixed in 4% (v/v) formaldehyde for 15 minutes at ambient temperature. Fixed cells were washed 3 times with PBS for 10 minutes, permeabilized with 0.2% Triton X-100 for 20 minutes, washed 3 more times, blocked in 1% bovine serum albumin (BSA) in PBS for 1 h, and incubated with primary antibodies in 0.1% BSA at 4 °C overnight. The cells were washed 3 times, incubated with secondary antibodies in 0.1% BSA for 2 h at ambient temperature, and the nuclei were counterstained with DAPI (0.1 μg/mL) for 10 minutes. Fluorescence images were acquired using a Nikon E600 inverted microscope. Primary antibodies were against: type VI collagen (rabbit clone, 1:100; Genetex, GTX109963), fibronectin (rabbit clone, 1:100; Novus Biologicals, NBP1-91258), pSMAD2/3 (rabbit clone, 1:100; R&D systems, MAB8935), type I collagen (mouse clone, 1:100; Abcam, ab6308), type II collagen (rabbit clone, 1:100; Abcam, ab34712), type III collagen (mouse clone, 1:100; Abcam, ab23445), or integrin β1 (rabbit clone, 1:100; Cell Signaling Technology, 34971S). Secondary antibodies were goat anti-mouse Alexa Fluor 647 or goat anti-rabbit Alexa Fluor 647 (both 1:500; Thermo Fisher Scientific).

### TGFβ1 ELISA Assay

TGFβ1 in the medium by hMSCs was quantified using a human TGFβ1 ELISA kit (antibodies online, ABIN625094). The medium was harvested on days 1, 2, 4, 6, 8, 12, and 14. After harvesting the medium, the cells were lysed for DNA quantification. The ELISA was performed according to the manufacturer’s instructions. Absorbance was measured at 450 nm using a ClarioStar plate reader (BMG LabTech). Background level of TGFβ1 in the growth medium without cells was subtracted from samples. Alternatively, TGFβ1 concentration was normalized to the total DNA content.

### DNA Quantification

After removing the medium, the hMSCs were washed twice with PBS and lysed with RLT lysis buffer (Qiagen). The lysate was freeze–thawed to ensure proper lysis. Samples were then diluted 50× in Tris-EDTA buffer (10 mM Tris-HCl, 1 mM EDTA, pH 7.5) and a DNA standard curve was made in the same final solution. A PicoGreen assay (Thermo Fisher Scientific) was used to quantify DNA, according to the manufacturer’s protocol. The fluorescence signal (excitation: 492 nm and emission: 520 nm) was obtained on a ClarioStar plate reader.

### EdU Cell Proliferation Detection

To assess the proliferation of hMSCs, 5-ethynyl-2ʹ-deoxyuridine (EdU) staining was conducted using the Click-iT EdU Alexa Fluor 647 Imaging Kit (Thermo Fisher Scientific). HMSCs were incubated with 50 μM EdU for 48 h before fixation in 4% (v/v) paraformaldehyde in PBS for 15 minutes at ambient temperature. Fixed samples were permeabilized with 0.2% Triton X-100 for 20 minutes, washed 3 more times, blocked in 1% BSA for 1 h, and the incorporated EdU was labeled with Alexa Fluor 647 azide for 30 minutes according to the manufacturer’s protocol. The nuclear DNA was counterstained by DAPI (0.1 μg/mL) for 30 minutes. Fluorescence images were acquired on a Nikon E600 inverted microscope.

### Statistics

The data are representative of at least 3 independent experiments with similar results. Statistics were determined using Student’s *t* test Statistics with *P*-values < .05 are considered significant. Statistical tests were performed with GraphPad Prism 8.

## Results

### Cadherin-11 is Necessary for Adipogenic Differentiation

**Figure 1.**
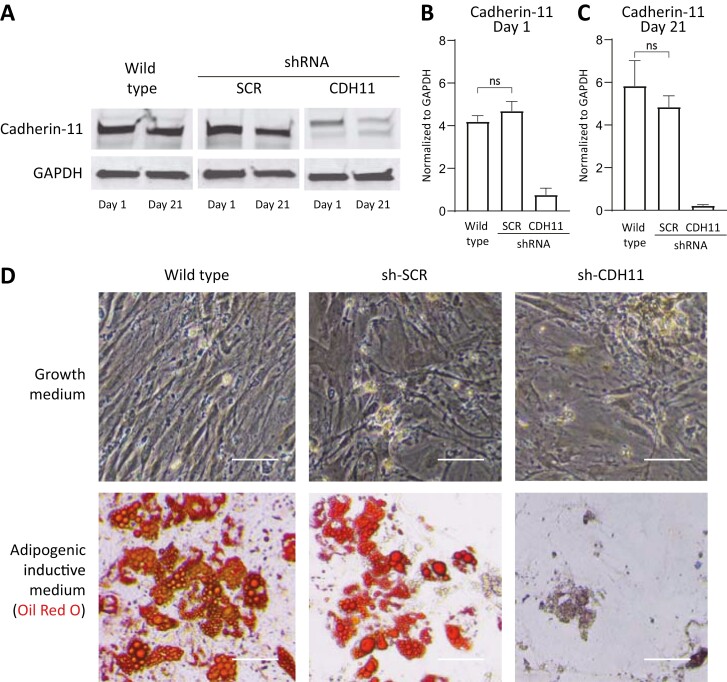
Loss of cadherin-11 disrupts the adipogenic potential of hMSCs. (**A**) Western blot analysis of cadherin-11 shows decreased expression in the cadherin-11-knockdown cells (sh-CDH11) compared with the wild type and scrambled (sh-SCR) controls at days 1 and 21. GAPDH is shown as a loading control. (**B**, **C**) Quantification of Western blots normalized to GAPDH showed that cadherin-11 expression in sh-CDH11 cells was significantly decreased compared with the wild type and sh-SCR controls at both days 1 and 21. Error bars show + SD. Statistical significance was determined using one-way ANOVA with Tukey’s test for multiple comparisons. All comparisons are statistically significant (*P* < .02) unless mentioned otherwise (n.s., not significant). (**D**) Brightfield micrographs of hMSCs stained with Oil Red O after 21 days in growth and adipogenic inductive medium show that sh-CDH11 cells had reduced lipid accumulation compared with wild-type control and sh-SCR, indicating disrupted adipogenic differentiation. Scale bars represent 100 μm. All data are representative of at least 3 independent experiments with similar results.

We performed lentiviral transduction using cadherin-11 or scrambled shRNA of hMSCs cultured in growth medium. Western blotting confirmed the knockdown of cadherin-11 on days 1 and 21 in culture (Fig. 1A). Quantification of the Western blot showed that the cadherin-11-knockdown cells had an 82% lower expression of cadherin-11 at day 1 (Fig. 1B) and 96% lower expression at day 21 (Fig. 1C) compared with the wild type. When subjected to adipogenic inductive medium for 21 days and stained with Oil Red O, we observed that cadherin-11-knockdown cells had reduced lipid accumulation compared to the scrambled and the wild-type cells (Fig. 1D). Similarly, when we quantified the lipid droplets stained with Oil Red O after 21 days in adipogenic inductive medium, we observed a significant decrease in Oil Red O absorbance in cadherin-11-knockdown cells compared to wild-type cells (*P < .*0001; Fig. S1).

### Cell Density Affects the Expression of Collagen

Since cadherin-11 has no known intrinsic signaling activity, we wanted to find evidence to explain how knocking down cadherin-11 leads to changes in the adipogenic differentiation potential of hMSCs. We first hypothesized that the loss of cadherin-11 could be affecting the expression of collagen, which is known to promote adipogenic differentiation.^16-18^ To test this hypothesis, we investigated various collagens that are expressed by hMSCs, namely type I collagen, type II collagen, type III collagen, and type VI collagen by immunofluorescence. At the same time, having observed that knocking down cadherin-11 caused a decrease in proliferation (Supplementary Fig. S2), we wanted to investigate if the changes in cell density could explain the changes in collagen expression. Among the various collagens tested, we observed that type I collagen, type II collagen, and type III collagen expression were cell density-dependent, namely we observed a higher intensity of staining in low-density cells (Supplementary Fig. S3A–C), meaning we could not attribute their expression directly to the cadherin-11 knockdown.

Out of curiosity, we also investigated type I collagen, type II collagen, and type III collagen on day 14. Type I collagen, type II collagen, and type III collagen express had no observable differences when comparing the cadherin-11-knockdown cells to the wild type at both days 1 and 14 (Supplementary Fig. S4A-C).

### Cadherin-11 Knockdown Decreases Type VI Collagen Expression

**Figure 2.**
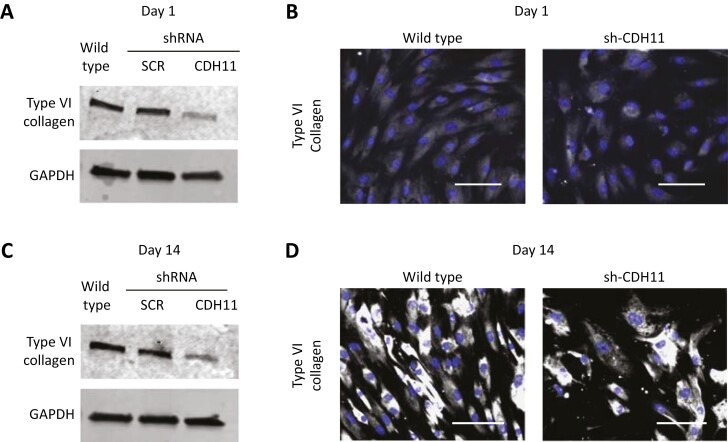
Cadherin-11 knockdown reduces the expression of type VI collagen. HMSCs were seeded at 1 × 10^4^ cells/cm^2^ and evaluated after 1 and 14 days. (**A**) Western blot analysis of type VI collagen on day 1 shows decreased expression in the cadherin-11-knockdown cells (sh-CDH11) compared with the wild type and scrambled (sh-SCR) controls. GAPDH is shown as a loading control. (**B**) Immunofluorescence micrographs of type VI collagen (white) on day 1 also show decreased expression in the sh-CDH11 cells compared with the wild type. (**C**) Western blot analysis on day 14 shows that the low type VI collagen expression persists in sh-CDH11 cells compared with the wild type and sh-SCR. (**D**) Immunofluorescence micrographs of type VI collagen (white) at day 14 confirm the decreased expression in sh-CHD11 cells compared with the wild type. Nuclei were counterstained with DAPI (blue). Data are representative of at least 3 independent experiments with similar results. Scale bars represent 100 μm.

When we tested the various collagens, we observed that type VI collagen expression remained unchanged over the different cell densities (Supplementary Fig. S5A). Furthermore, type VI collagen is highly enriched in the ECM of adipocytes,^19^ leading us to investigate it further. When we performed Western immunoblotting, we observed that cadherin-11-knockdown cells had significantly lower levels of type VI collagen when compared with the wild type at day 1 (Fig. 2A and Supplementary Fig. S6A). Immunofluorescence micrographs at day 1 confirmed the decrease in type VI collagen in cadherin-11-knockdown cells (Fig. 2B). Since collagen production increases over time, we also investigated the expression of type VI collagen on day 14. Western blot analysis showed a significant decrease in type VI collagen at day 14 compared with the wild type (Fig. 2C and Supplementary Fig. S6B). This decrease in type VI collagen expression in the cadherin-11-knockdown cells was confirmed by immunofluorescence micrographs (Fig. 2D).

### Cadherin-11 Knockdown Increases Fibronectin Expression

**Figure 3.**
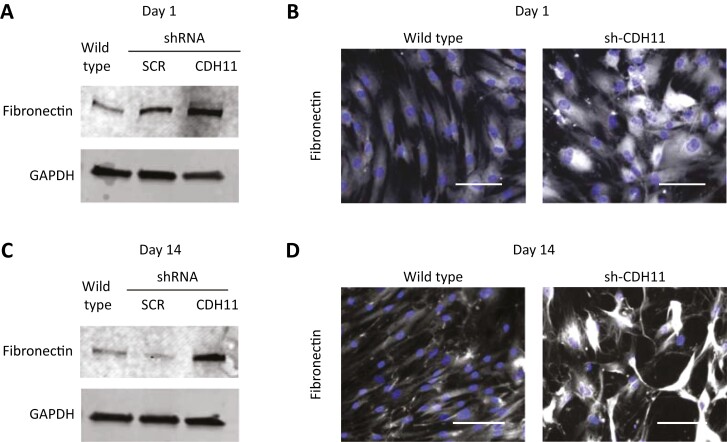
Cadherin-11 knockdown reduces the expression of fibronectin. HMSCs were seeded at 1 × 10^4^ cells/cm^2^ and evaluated after days 1 and 14. (**A**) Western blot analysis of fibronectin on day 1 shows increased expression in cadherin-11-knockdown cells (sh-CDH11) compared with the wild type and scrambled (sh-SCR) controls. GAPDH is shown as a loading control. (**B**) Immunofluorescence micrographs of fibronectin (white) at day 1 also show increased expression in the sh-CDH11 cells compared with the wild type. (**C**) Western blot analysis on day 14 shows that the fibronectin expression persists in sh-CDH11 cells compared with the wild type and sh-SCR. (**D**) Immunofluorescence micrographs of fibronectin (white) at day 14 confirm the increased expression compared to the wild type. Nuclei were counterstained with DAPI (blue). Data are representative of at least 3 independent experiments with similar results. Scale bars represent 100 μm.

Given our observation of reduced type VI collagen expression, we sought to investigate if the cadherin-11 knockdown affected the expression of other ECM proteins. We first confirmed that the expression of fibronectin was not influenced by cell density (Supplementary Fig. S5B). Next, we investigated the expression of fibronectin at day 1 and observed a significant increase in cadherin-11-knockdown cells compared with the wild type (Fig. 3A and Supplementary Fig. S7A). Immunofluorescence micrographs confirmed the increase in fibronectin in cadherin-11-knockdown cells compared with the wild type at day 1 (Fig. 3B). We also observed a change in the expression pattern of fibronectin between the wild type and cadherin-11-knockdown cells, where the cadherin-11-knockdown cells had enriched fibronectin surrounding the cell, while the wild-type cells had fibronectin expression throughout their ECM (Fig. 3B).

We then investigated integrin β1 to confirm the differences in the fibronectin pattern and we observed it closely followed the pattern of fibronectin (Supplementary Fig. S6). We also performed a Western blot after 14 days in culture and observed that cadherin-11-knockdown cells had significantly increased fibronectin expression compared with the wild type (Fig. 3C and Supplementary Fig. S7B). Again, on day 14, immunofluorescence micrographs confirmed the increase in fibronectin in cadherin-11-knockdown cells (Fig. 3D).

### Increased Nuclear Localization of Phosphorylated Smad2/3 in Cadherin-11-knockdown Cells

To better understand how the loss of cadherin-11 changed the ECM composition of hMSCs, we looked at TGFβ1, a well-known inducer of ECM components such as collagen and fibronectin.^20^ Given that the TGFβ1 pathway is implicated in ECM regulation, we hypothesized that cadherin-11-knockdown cells have enhanced TGFβ1 secretion which therefore changes the ECM.

**Figure 4.**
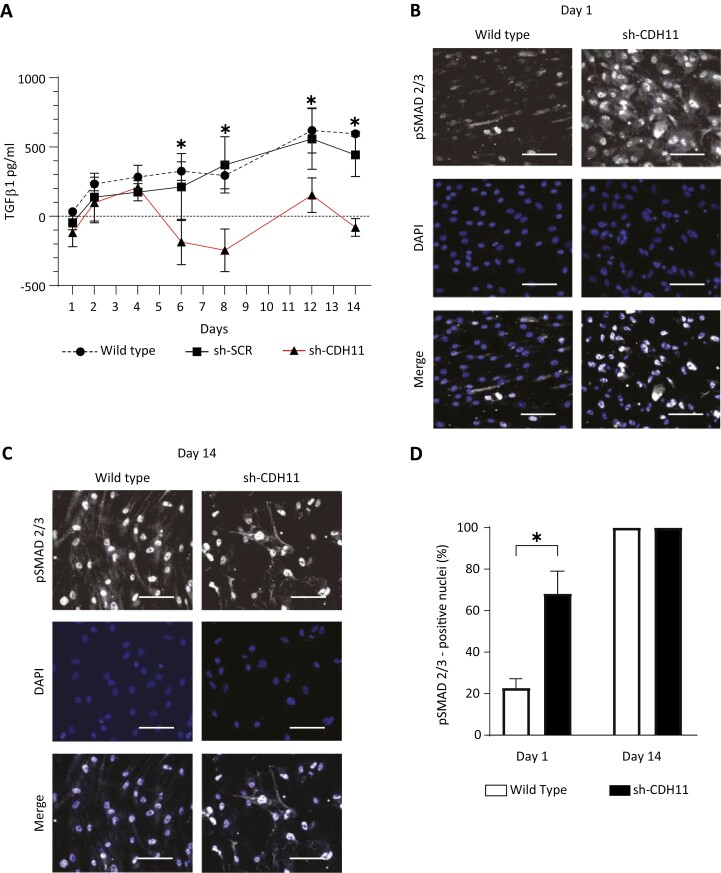
Cadherin-11-knockdown cells have more pSMAD2/3-positive nuclei. (**A**) Time course of TGFβ1 in the medium of hMSCs, where medium was collected and total TGFβ1 was measured using ELISA following acidification. The baseline level of TGFβ1 in the medium without cells is indicated with a dashed line. Compared with wild type and scrambled control (sh-SCR), less TGFβ1 was detected in the supernatant in cadherin-11-knockdown cells (sh-CDH11). Statistics were determined using 2-way ANOVA with Tukey's test for multiple comparisons. *P < .001, sh-CDH11 compared with both wild type and sh-SCR. Error bars show ± SD. Data are representative of at least 3 independent experiments with similar results. Immunofluorescence micrographs at (**B**) day 1 and (**C**) day 14 of hMSCs seeded at 1 × 10^4^ cells/cm^2^ and immunostained for pSMAD2/3 (white) and counterstained with DAPI (blue). Scale bars represent 100 μm. (**D**) Quantification of the number of positive pSMAD 2/3 nuclei shows cadherin-11-knockdown (sh-CDH11) cells have more pSMAD2/3-positive nuclei compared with the wild type at day 1. Statistics were determined using Student's *t*-test: *P < .003.

We first wanted to investigate if cadherin-11-knockdown cells indeed secreted more TGFβ1 compared with the wild type. To this end, we performed TGFβ1 ELISA on the supernatant collected from the cell culture at days 1, 2, 4, 6, 8, 12, and 14. We subtracted the baseline levels of TGFβ1 present in the medium without cells from the level of TGFβ1 measured at a particular time point. We observed an increase in TGFβ1 from a mean value of 33 pg/mL at day 1 to 595 pg/mL at day 14. There was no significant difference observed in the values between the wild type and the scrambled controls, but TGFβ1 in the cadherin-11-knockdown cells had a mean value of −118 pg/mL at day 1 and −80 pg/mL at day 14, implying that there was less in the supernatant than in the medium without cells. After 6 days in culture, the amount of TGFβ1 measured in the supernatant of the cadherin-11-knockdown cells was significantly lower than both the wild type and the scrambled controls (Fig. 4A; *P < .*0001). This trend persisted at days 8, 12, and 14 (Fig. 4A; *P < .*0002). We also normalized the same data of TGFβ1 quantity to the DNA content, revealing that the average amount of TGFβ1 per cell in the cadherin-11-knockdown cells was significantly higher than in the wild-type cells from day 4 onward (Supplementary Fig. S6; *P < .*03).

Since the levels of TGFβ1 themselves were insufficient to explain our observations about the ECM, we looked further at SMAD2/3, which is the downstream signaling molecule of the TGFβ1 pathway. TGFβ1 stimulation leads to phosphorylation and activation of SMAD2/3, which accumulates in the nucleus and regulates the transcription of ECM-related genes. On day 1, we observed that wild-type cells had fewer nuclei positive for phosphorylated SMAD2/3 (pSMAD2/3) compared with the cadherin-11-knockdown cells, indicating that the pathway was not active in the wild type but was active in the cadherin-11-knockdown cells (Fig. 4B). When this was quantified, the wild-type cells had 22.8 ± 5% nuclei positive for pSMAD2/3 compared with 68.2 ± 10% in cadherin-11-knockdown cells (Fig. 4D; *P < .*0001). After 14 days in culture, all nuclei were positive for pSMAD2/3 in both the wild-type and cadherin-11-knockdown cells (Fig. 4C,D).

## Discussion

The importance of the ECM in influencing cell behavior is unquestionable. Our study shows that cadherin-11 influences cell differentiation indirectly by regulating the ECM via the TGFβ1 pathway. Previous studies including ours have implicated cadherin-11 in osteogenic, smooth muscle cell, and adipogenic differentiation.^4,5^ So far, the only evidence for signaling events involving cadherin-11 has been provided for smooth muscle cell differentiation.^5^ Notably, cadherin-11 does not have any known intrinsic signaling activity, but our work reveals how knocking it down inhibits the differentiation of hMSCs toward the adipogenic lineage (Fig. 1).

We were inspired by a study that showed that cadherin-11 was necessary for ECM production in fibroblasts and smooth muscle–containing tissue.^12^ The authors discovered that cadherin-11^−/−^ mice had significantly reduced type I collagen, type III collagen, and elastin expression.^12^ In order to say whether cadherin-11 influences the ECM of hMSCs, we first screened a selection of ECM components. We started by looking at collagens, as they are the most abundant ECM constituent, and compared their expression in the cadherin-11-knockdown cells to the wild-type cells. Since the cadherin-11 knockdown causes the cells to proliferate more slowly (Supplementary Fig. S1) we chose to investigate ECM proteins with expression that was independent of cell density. We discovered that cadherin-11-knockdown cells had decreased expression of type VI collagen but an increased expression of fibronectin (Fig. 2,3).

Adipogenic differentiation is usually associated with ECM remodeling, characterized by the conversion from the fibronectin and type I collagen matrix to laminin and type VI collagen.^19,21^ Our current understanding of the function of type VI collagen comes mainly from the study of a mouse model with a defective type VI collagen gene, which leads to muscle myopathy that progresses with age.^22,23^ A recent study found that mature adipocyte differentiation was attenuated in type VI collagen-deficient cells.^24^ Therefore, the reduced expression of type VI collagen in cadherin-11-knockdown cells is related to the reduced capacity for adipogenic differentiation. Similarly, the growth of preadipocytes on a fibronectin matrix is found to inhibit adipocyte differentiation.^25^ Therefore, the increased expression of fibronectin in cadherin-11-knockdown cells is likely to be related to their reduced capacity for adipogenic differentiation.

TGFβ1, a potent and pleiotropic cytokine in its biologically active form, binds to its receptor and stimulates expression of ECM components via phosphorylation of the signaling molecules SMAD2/3.^26-28^ We speculated that the TGFβ1 pathway may be differentially regulated in cadherin-11-knockdown cells, resulting in changes in ECM, and we, therefore, investigated the expression pSMAD2/3. In our study, we provide evidence of decreased TGFβ1 in the supernatant and the subsequent translocation of pSMAD2/3 into the nucleus by cells that lack cadherin-11, which could be related to the changes in ECM composition (Fig. 4). The cadherin-11 expression has been shown to be upregulated by exogenous TGFB1 supplementation in myofibroblasts.^29^ In our study, we showed that the TGFβ1 pathway is upregulated earlier in cadherin-11-knockdown cells compared with the wild type. TGFβ1 has also been linked to suppressed adipogenic differentiation by hMSCs when it is supplemented before commitment.^30-32^ Seeing as the TGFβ1 pathway is activated prematurely in cadherin-11-knockdown cells, it is possible that a similar mechanism is observed.

Fibronectin binds a plethora of growth factors that are central in tissue repair and fibrosis, including latent TGFβ.^33,34^ Seeing as pSMAD2/3 is already translocated into the nucleus at day 1, the rest of the TGFβ1 probably remains bound to the ECM, which is why we saw a decrease inTGFβ1 over time. Another explanation is that hMSCs secrete TGFβ1 in the presence of cadherin-11, as knocking down cadherin-11 reduced the levels of TGFβ1. Mouse MSCs are known to secrete TGFβ and alveolar macrophages isolated from the lungs of cadherin-11-deficient mice exhibit lower levels of TGFβ.^5,10,35^ However, these studies have linked low levels of TGFβ with a decrease in pSMAD2/3, which is not in line with our study.

Given the increase in fibronectin levels in cadherin-11-knockdown cells, we suggest that there could be an involvement of integrin signaling. Integrins α5 and α6 have been implicated in adipogenic differentiation and fibronectin acts as a ligand for dozens of the integrin family members.^36,37^ While we only looked at the patterning of integrin β1 and compared it with the fibronectin expression, there is a need for studies that look at the changes to other integrin complexes. Further studies are needed to investigate whether the change in integrin β1 patterning was due to the changes in the ECM or irrespective of it.

Cadherin-11 is known to forms a complex with certain transmembrane receptors.^38^ In this study, we make our conclusions based on lentiviral knockdowns. Using a small molecule to inhibit cadherin-11 could potentially result in different observations as the currently available small molecule inhibitors for cadherin-11 bind to the extracellular domain of the cadherin and only prevent cadherin-cadherin interaction. Furthermore, future studies using RNA sequencing techniques as well as computational modeling could give us an insight into the multilayer crosstalk that exists between cadherin-11 and other signaling pathways.

## Conclusion

Taken together, our study demonstrates that cadherin-11 regulates the ECM by temporally controlling the TGFβ pathway. This improves the understanding of hMSC fate commitment and adds evidence to the importance of cadherin-11 in the differentiation of hMSCs.

## Supplementary Material

sxac026_suppl_Supplementary_Figures

## Data Availability

The data that support the findings of this study are available from the corresponding author upon reasonable request.
