# Supplementary material for: Cadherin-11 Influences Differentiation in Human Mesenchymal Stem Cells by Regulating the Extracellular Matrix Via the TGFβ1 Pathway
Source: Stem Cells. 2022 Apr 13;40(7):669–77. doi: 10.1093/stmcls/sxac026 (PMC9332898; doi:10.1093/stmcls/sxac026)
Supplement: sxac026_suppl_Supplementary_Figures [file sxac026_suppl_supplementary_figures.docx]

**SUPPLEMENTARY FIGURE 1**

**
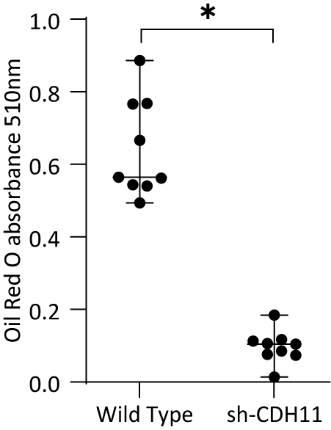
**

**Supplementary Figure 1:** Cadherin-11–knockdown cells have significantly reduced lipid accumulation. Quantification of the lipid droplets stained with Oil Red O after 21 days in the adipogenic inductive medium by measuring absorbance at 510 nm. The data were normalized to values of non-induced control. Error bars show ± SD. N=3. Statistics were determined using Welch's t-test: **p*< 0.0001.

**SUPPLEMENTARY FIGURE 2**


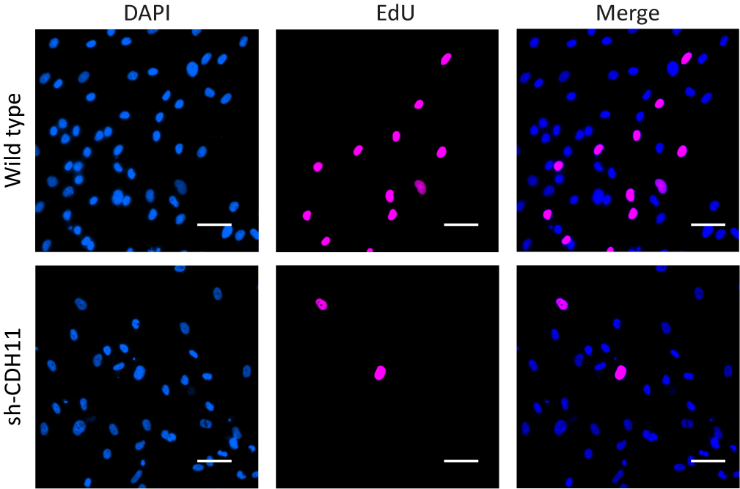


**Supplementary Figure 2: Cadherin-11–knockdown cells have reduced proliferation.**

Immunofluorescence micrographs of hMSCs seeded at 1 × 10^4^ cells/cm^2^ show reduced EdU in cadherin-11–knockdown (sh-CDH11) cells after 2 days in culture. DAPI (blue) and EdU (magenta) staining of hMSCs incubated with EdU for 48 h in growth medium. Data are representative of at least three independent experiments with similar results. Scale bars represent 100 μm.

**SUPPLEMENTARY FIGURE 3**


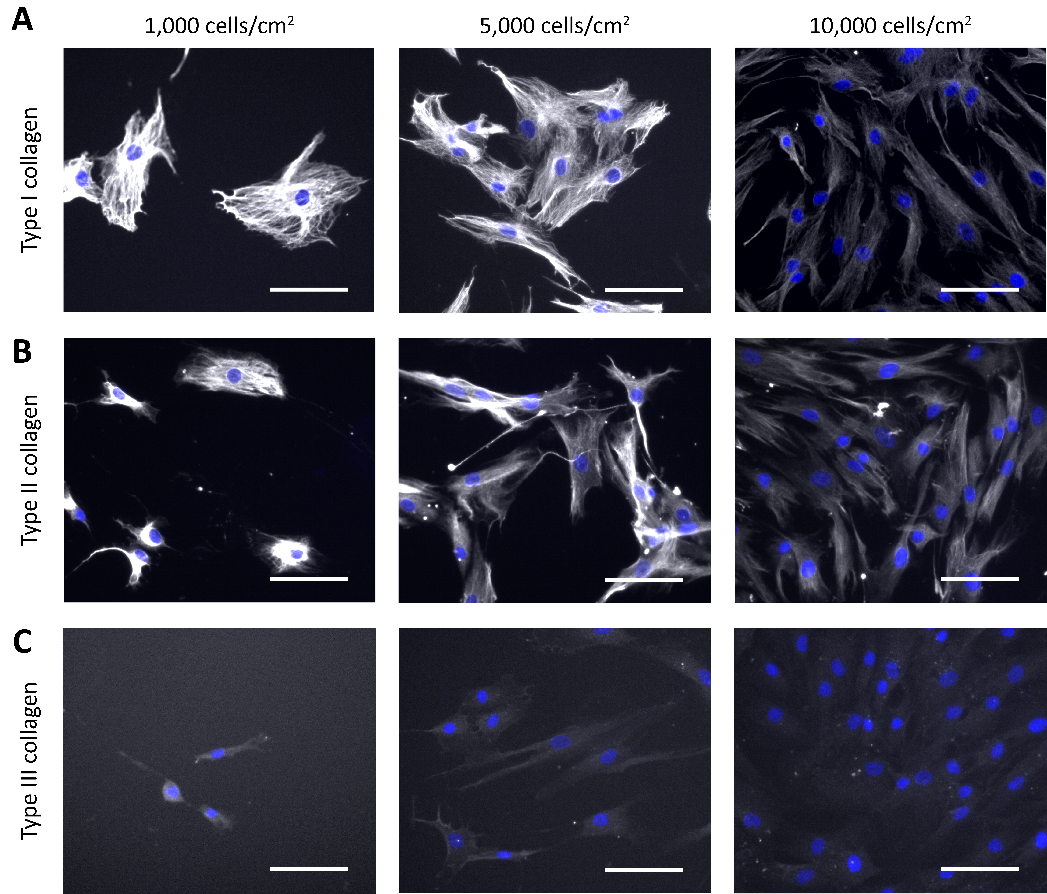


**Supplementary Figure 3: Type I, II and III collagen expression decrease with increasing cell density**. Immunofluorescence micrographs of hMSCs seeded at different densities: 1 × 10^3^ cells/cm^2^, 5 × 10^3^ cells/cm^2^, and 1 × 10^4^ cells/cm^2^ and evaluated 24 h after seeding. (A) Type I collagen (white) expression decreases with increasing cell density. (B) Type II collagen (white) expression decreases with increasing cell density. (C) HMSCs have a low expression of type III collagen (white) which decreases with increasing density. Nuclei are stained with DAPI (blue). Data are representative of at least three independent experiments with similar results. Scale bars represent 100 μm.

**SUPPLEMENTARY FIGURE 4**

**
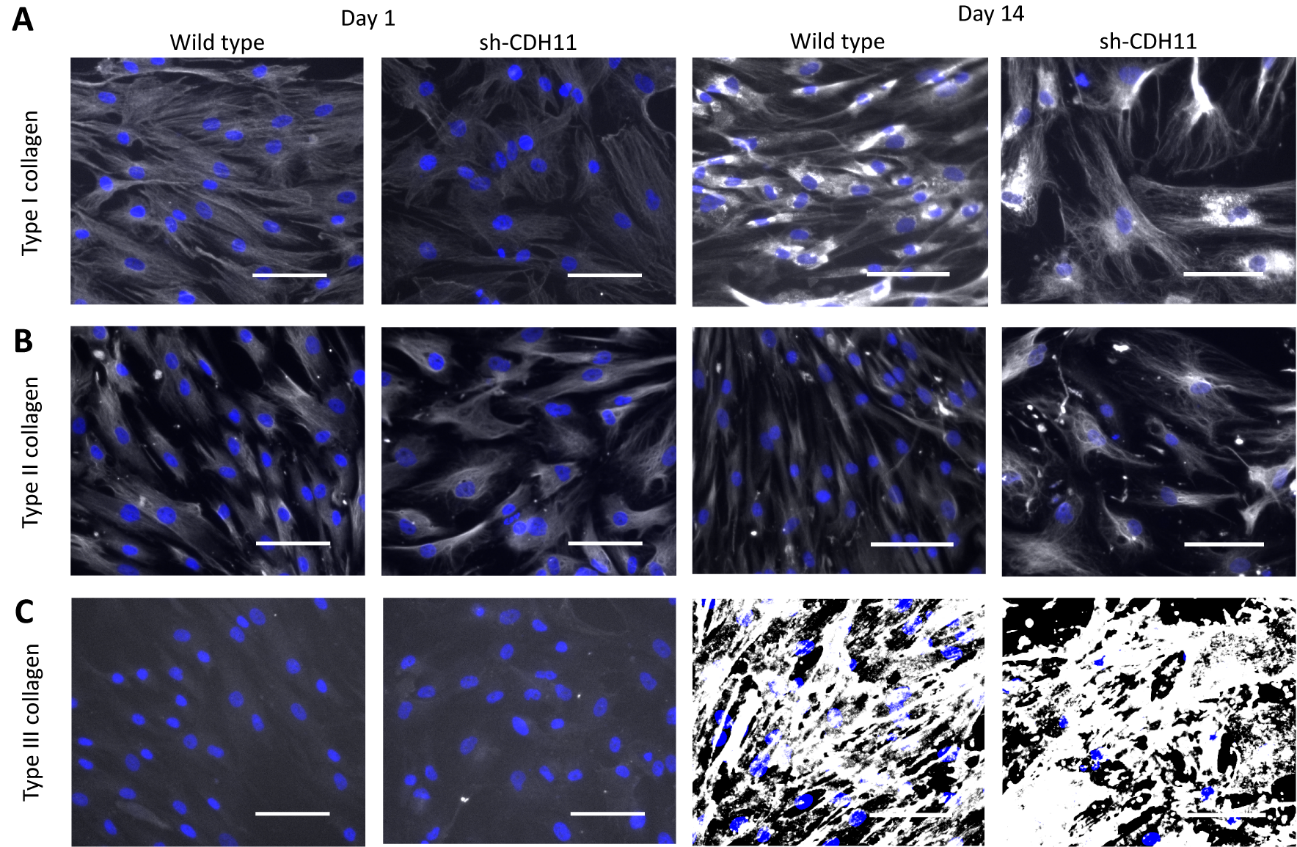
**

**Supplementary Figure 4: Type I, II, and III collagen expression in cadherin-11–knockdown cells.** Immunofluorescence micrographs of hMSCs seeded at 1 × 10^4^ cells/cm^2^ and evaluated after days 1 and 14. (A) No difference in type I collagen (white) expression was observed in wild type and sh-CDH11 cells.. (B) No difference in type II collagen (white) expression was observed in wild type and sh-CDH11 cells. (C) No difference in type III collagen (white) expression was observed in wild type and sh-CDH11 cells. Nuclei are stained with DAPI (blue). Data are representative of at least three independent experiments with similar results. Scale bars represent 100 μm.

**SUPPLEMENTARY FIGURE 5**


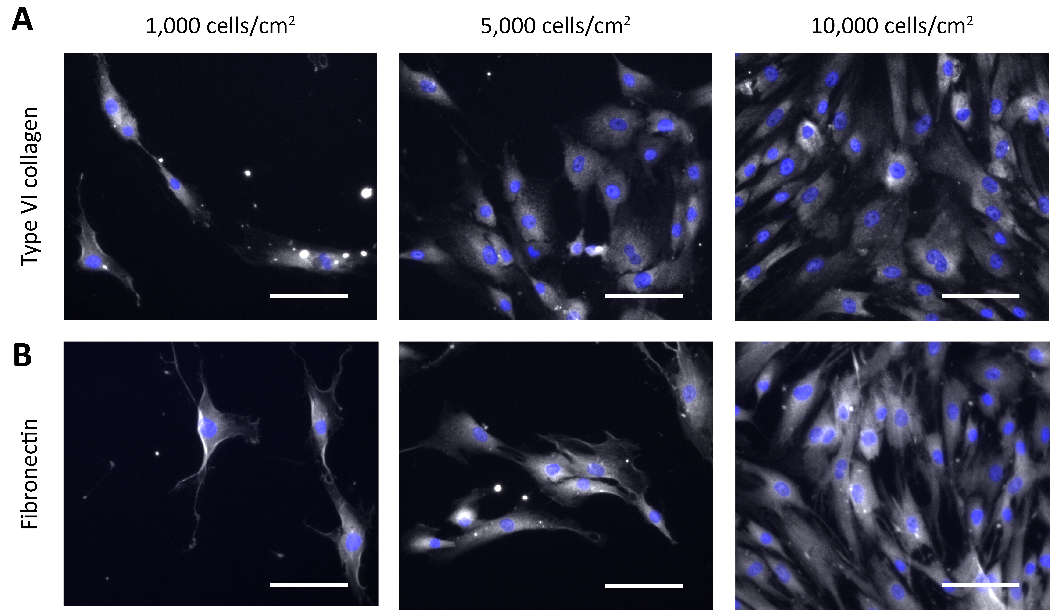


**Supplementary Figure 5: Type VII collagen and fibronectin expression remain unchanged with increasing cell density.** Immunofluorescence micrographs of hMSCs seeded at different densities: 1 × 10^3^ cells/cm^2^, 5 × 10^3^ cells/cm^2^, and 1 × 10^4^ cells/cm^2^. (A) Type VI collagen (white) expression remains unchanged with increasing density. (B) Fibronectin expression (white) remains unchanged with increasing density. Nuclei are stained with DAPI (blue). Data are representative of at least three independent experiments with similar results. Scale bars represent 100 μm.

**SUPPLEMENTARY FIGURE 6**

**
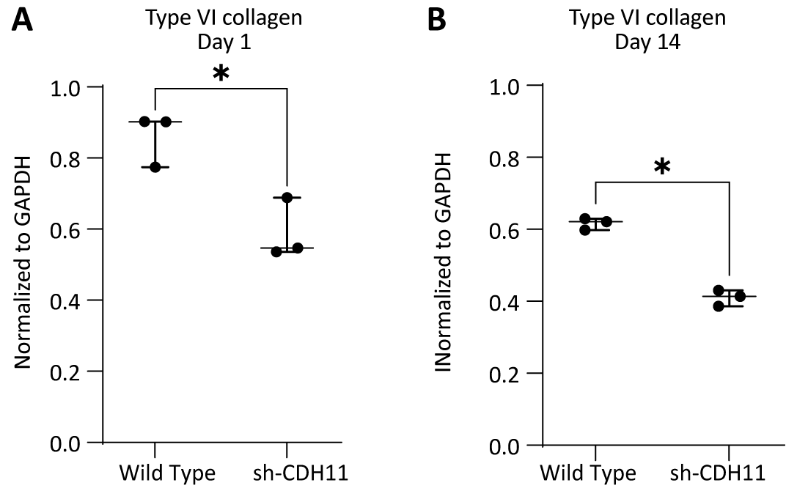
**

**Supplementary Figure 6:** Cadherin-11–knockdown decreases type VI collagen expression. Quantification of Western blots normalized to GAPDH showed that type VI collagen expression significantly decreased at (A) Day 1 and (B) Day 14. N=3. Statistics were determined using Student’s t-test: *p<0.01.

**SUPPLEMENTARY FIGURE 7**

**
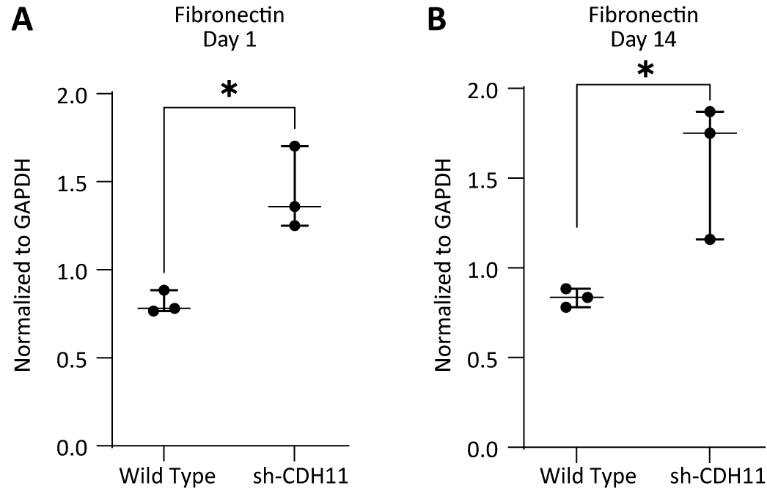
**

**Supplementary Figure 7:** Cadherin-11–knockdown increases fibronectin expression. Quantification of Western blots normalized to GAPDH showed that fibronectin expression significantly increased at (A) Day 1 and (B) Day 14. N=3. Statistics were determined using Student’s t-test: *p<0.02.

**SUPPLEMENTARY FIGURE 8**


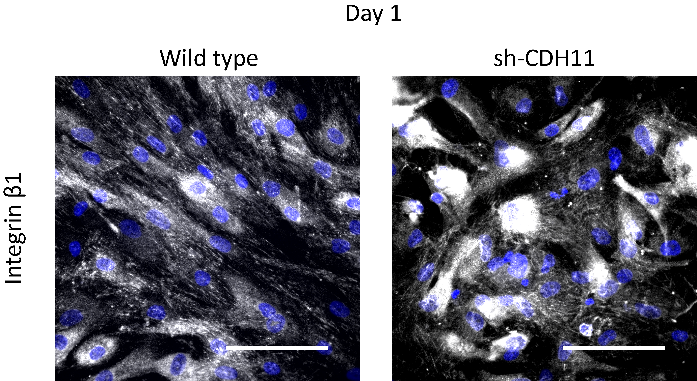


**Supplementary Figure 8:** **Integrin β1 followed the pattern of fibronectin.** Immunofluorescence micrographs of hMSCs seeded at 1 × 10^4^ cells/cm^2^. Integrin β1 (white) follows a pattern similar to the fibronectin. Nuclei are stained with DAPI (blue). Data are representative of at least three independent experiments with similar results. Scale bars represent 100 μm.

**SUPPLEMENTARY FIGURE 9**


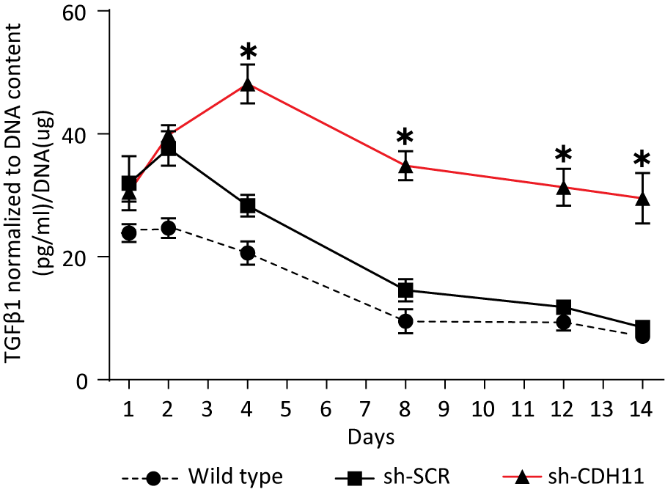


**Supplementary Figure 9: More TGFβ1 in the medium of cadherin-11–knockdown cells.**  Time course of TGFβ1 in the medium of hMSCs, where medium was collected and total TGFβ1 was measured using an ELISA following acidification. The data were normalized to the DNA content (proportional to cell number) in each sample (n=3). Compared to wild type and scrambled control (sh-SCR), less TGFβ1 was detected in the supernatant in cadherin-11–knockdown cells (sh-CDH11). Statistics were determined using two-way ANOVA with Tukey's test for multiple comparisons: **p*< 0.03, sh-CDH11 compared to both wild type and sh-SCR. Error bars show ± SD. Data are representative of at least three independent experiments with similar results.
